# Rhodococcus Empyema in an Immunocompetent Patient

**DOI:** 10.7759/cureus.48540

**Published:** 2023-11-08

**Authors:** Joanna Wieckowska, Shannon Hood, Dalia Zakri, Mazen Najjar

**Affiliations:** 1 Pulmonary and Critical Care, Ascension Health, Grand Blanc, USA; 2 Graduate Medical Education, Michigan State University School of Osteopathic Medicine, Lansing, USA; 3 Infectious Disease, Ascension Health, Grand Blanc, USA

**Keywords:** case report, rare, immunocompetent, empyema, rhodococcus equi, rhodococcus hoagii

## Abstract

*Rhodococcus (R.) equi* is a gram-positive, facultative intracellular coccobacilli that most commonly causes pulmonary infections in animals and, rarely, in immunocompromised humans. Infected patients typically experience severe pulmonic infections such as necrotizing or cavitary pneumonia. We describe a rare case of *R. equi* that was isolated from an empyema in a 66-year-old immunocompetent patient experiencing recurrent pleural effusions requiring multiple interventions.

## Introduction

*Rhodococcus (R.)* ​​​​​*equi* is an intracellular aerobic, Gram-positive, weakly acid-fast coccobacillus. It is a common cause of infection in animals and a rare cause in humans. Most cases occur in immunocompromised patients (e.g., HIV/AIDS, solid organ and hematopoietic stem cell transplant recipients, lymphoma, cancer, prolonged steroid use) and only a limited number of cases have reported infection in immunocompetent patients. Rhodococcus infection commonly presents as severe pneumonia and may be complicated by abscesses, empyema, pleural effusion, and spontaneous pneumothorax [1-6]. A case of necrotizing pneumonia due to *R. equi* has also been reported [7]. We present a rare case of *R. equi* empyema in an immunocompetent patient.

## Case presentation

The patient was a 66-year-old male with a medical history significant for chronic obstructive pulmonary disease (COPD), congestive heart failure, testicular cancer status post resection (2013), former smoker with 45-pack-years, and recurrent pleural effusions status post multiple thoracenteses and video-assisted thoracoscopic surgery (VATS) from which cytology and flow cytometry were negative for malignancy with eventual CareFusion PleurX Pleural Catheter (McGaw Park, IL, USA) placement. He presented to the hospital for progressively worsening shortness of breath and nonproductive cough. His PleurX stopped draining several days ago.

**Figure 1:**
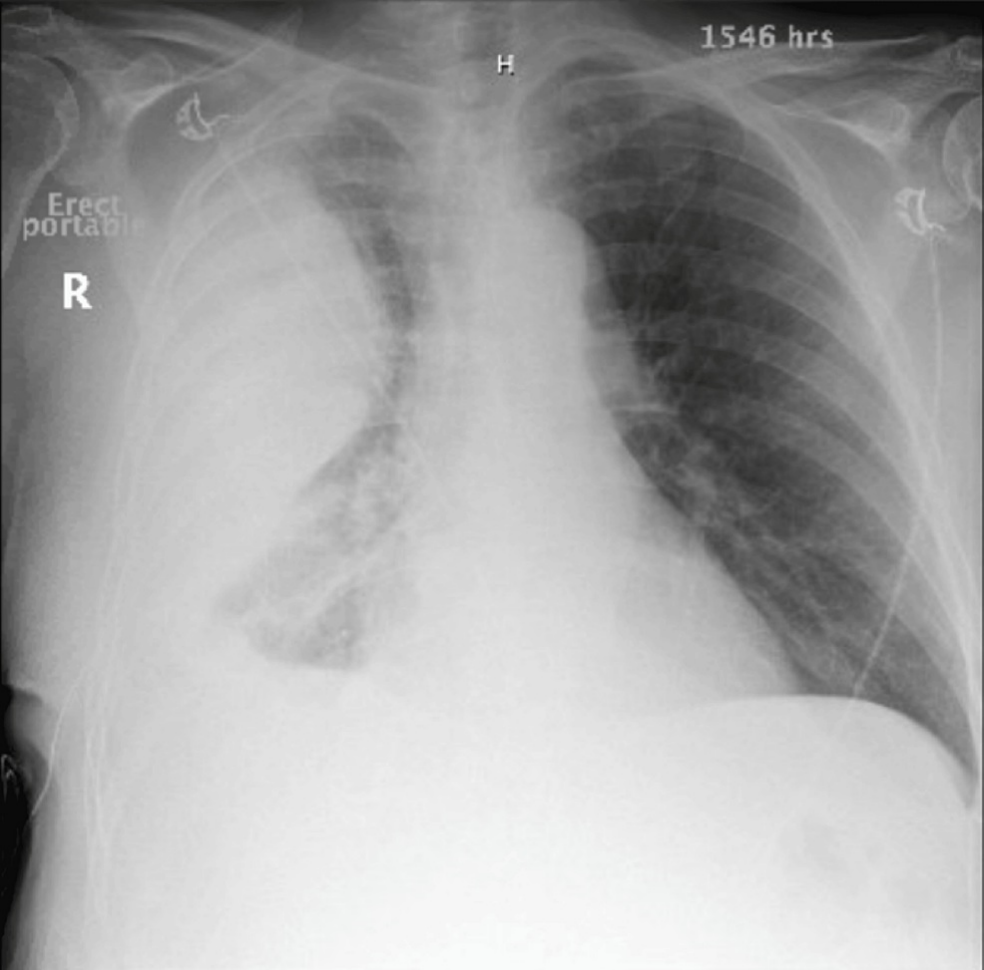
AP CXR shows a large loculated right pleural effusion with a drastic interval increase AP: anteroposterior; CXR: chest X-ray

**Figure 2:**
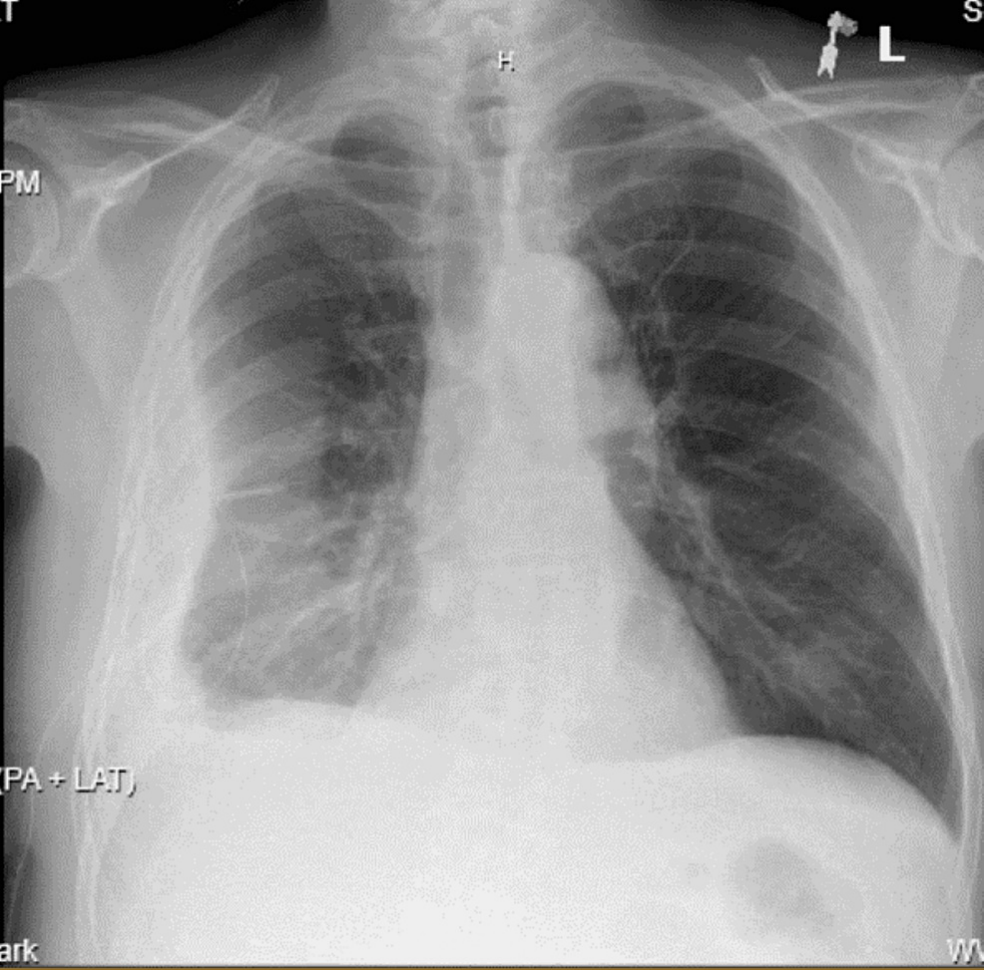
AP CXR one month prior to hospitalization shows a small partially loculated right pleural effusion with the chest tube in the appropriate position and hazy atelectasis versus infiltrate in the mid to lower right lung AP: anteroposterior; CXR: chest X-ray

**Figure 3:**
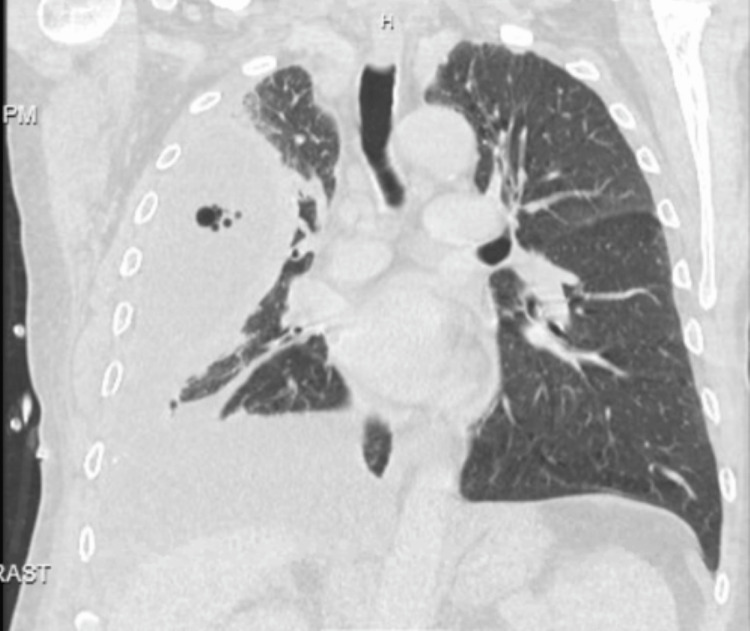
CT chest. Coronal view Showing a large, loculated right pleural effusion with the right chest drain in the appropriate position. Air pockets in the fluid could be related to the presence of the chest drain or could represent an infectious process. There is dense compressive atelectasis of the right lung and moderate left pleural effusion.

**Figure 4:**
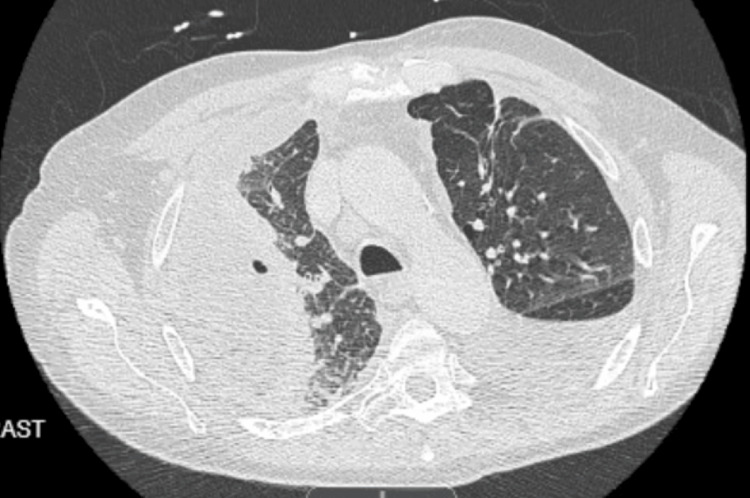
CT chest. Axial view Showing a large, loculated right pleural effusion with the right chest drain in the appropriate position. Air pockets in the fluid could be related to the presence of the chest drain or could represent an infectious process. There is dense compressive atelectasis of the right lung and moderate left pleural effusion.

On presentation, he required bilevel positive airway pressure (BiPAP) but was later successfully weaned to a nasal cannula. Chest radiography (CXR) showed a very large, loculated, right-sided pleural effusion with a drastic interval increase from one month ago (Figures 1, 2). Computed tomography (CT) chest re-demonstrated the large, loculated, right-sided pleural effusion seen on CXR along with air pockets possibly related to the presence of the chest drain or an infectious process, dense compressive atelectasis of the right lung, and moderate left pleural effusion (Figures 3, 4). The patient was started on intravenous vancomycin and piperacillin-tazobactam.

**Figure 5:**
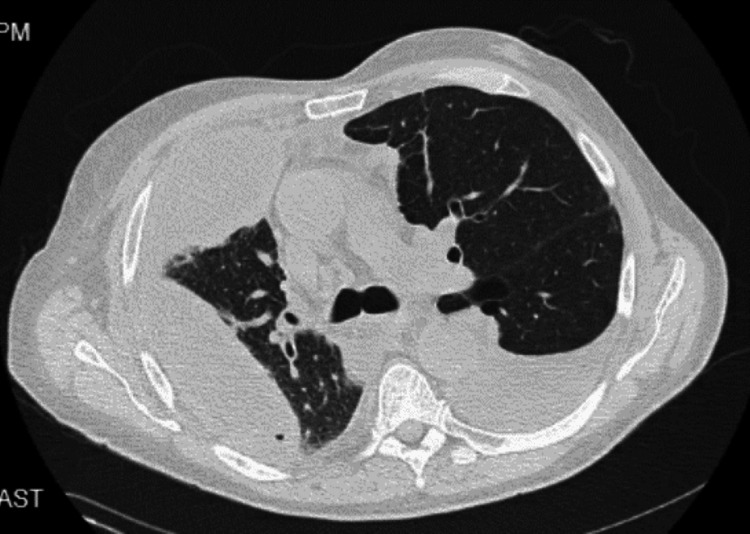
CT chest. Axial view (Contd.) Showing improved but persistent moderate loculated right pleural fluid with tiny foci of air with a chest drain within the inferior loculation

**Figure 6:**
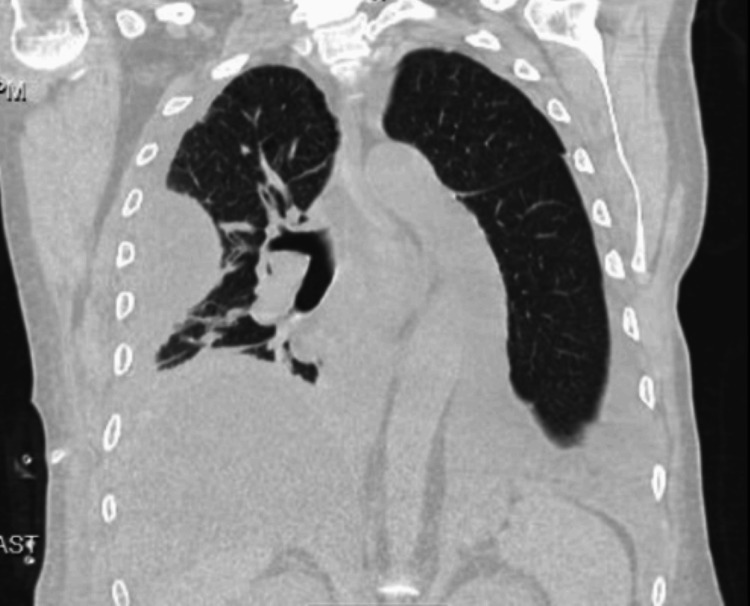
CT chest. Coronal view (Contd.) Showing improved but persistent moderate loculated right pleural fluid with tiny foci of air with a chest drain within the inferior loculation

Cardiothoracic surgery (CTS) was consulted and after some manipulation of the PleurX catheter, they instilled intrapleural tissue plasminogen activator (tPA) and dornase alfa (DNase) to help break up the pleural loculations with 3100 mL output. This pleural fluid was sent for culture and ultimately resulted in no growth. The decision was then made to drain the catheter every other day and discontinue antibiotics. Over the next few days, tPA DNase was instilled a total of six times but with minimal output. Shortly thereafter, the patient again began experiencing shortness of breath with new intermittent fevers and leukocytosis. He was restarted on broad-spectrum antibiotics, this time with intravenous cefepime and doxycycline. Repeat CT showed persistent right-sided loculated effusion (Figures 5, 6). Infectious disease (ID) was consulted, which recommended a second pleural fluid analysis, obtained a few days after the initial culture, which preliminarily grew Gram-positive rods. As this was now concerning for empyema, ID switched antibiotics to intravenous vancomycin and ampicillin-sulbactam, to account for broader gram-positive and anaerobic coverage. Within 48 hours, this second pleural fluid culture appeared to have grown diphtheroids on Gram stain. However, ID requested that this gram-positive rod resembling diphtheroids be sent to the State Department of Health laboratory for further identification due to the patient’s ongoing sepsis.

As the patient continued to experience ongoing sepsis for several days with concerns for empyema, it was decided to remove the PleurX catheter and perform a re-do VATS. The patient underwent right VATS with total pulmonary decortication, washout, chest tube placement, and PleurX removal without complications. Not long after, the pleural fluid culture that was sent to the State Department of Health finalized and grew *R. equi*. At this point, the patient had been hospitalized for at least one month and had received multiple, consecutive courses of intravenous antibiotics. After he underwent surgical intervention, he began to clinically improve and his O_2_ supplementation was weaned to room air. Upon hospital discharge and after much discussion, ID prescribed long-term treatment with levofloxacin and rifampin for a total of three months with recommendations for close outpatient follow-up.

## Discussion

*R. equi* is a Gram-positive, facultative intracellular coccobacilli that replicates within macrophages. First identified in 1923, *R. equi* was found to be infecting foals with granulomatous pneumonia and lung abscesses [8,9]. This microorganism is a well-known bacteria in the veterinary medicine world, as it is typically found in the soil, and especially in environments involving animals such as foals and pigs [8]. It was not until 1967 that *R. equi* presented in humans [1,9]. Although rare in humans, when cases do occur, they most frequently occur in those who are immunocompromised, such as transplant recipients and those with HIV/AIDS, making *R. equi* an opportunistic pathogen [1,6].

Once *R. equi* enters the body, whether through inhalation of contaminated soil, ingestion, or contamination of open wounds, it can infect almost any area from superficial wounds to joints, the CSF, and most often, the respiratory tract [1,9,10]. Patients may present with typical symptoms of pneumonia, such as a productive cough, fever, fatigue, and hemoptysis. Suspicion for *R. equi* should be high in those with necrotizing pneumonia or ill-defined, irregular, upper lobe cavitary lesions on chest imagining and especially in those with recreational or occupational exposure to large animals [11]. A definitive diagnosis is obtained with a culture of the organism from the source of infection [1]. In cases of pulmonary infections, cultures can be obtained from sputum samples, bronchial washings, tissue samples, or, as in the case of our patient, pleural fluid. However, diagnosis of *R. equi* can be difficult because of its diphtheroid morphology on Gram stain and positive acid-fast, periodic acid-Schiff (PAS) and Gomori methenamine silver (GMS) stains. It is commonly mistaken for a diphtheroid contaminant or a mycobacterium based on its acid-fast appearance, which often leads to delayed or missed diagnosis [12,13]. If our ID physician had not sent the pleural fluid culture results for further analysis to the State Department of Health laboratory, our patient may have also fallen victim to further delay in diagnosis based on the initial results.

Since *R. equi* easily infects those who are immunocompromised, infections diagnosed in immunocompetent patients should raise clinical suspicion for underlying immunodeficiency. Our workup to rule out an immunodeficiency and autoimmune disease in our patient, including quantitative immunoglobulins levels, antinuclear antibody (ANA), rheumatoid factor, anti-cyclic citrullinated peptide (CCP), anti-double-stranded deoxyribonucleic acid (dsDNA), anti-ribonucleoprotein (RNP), anti-Smith, anti-Sjogren's Sjogren's syndrome A (SSA) and Sjögren's syndrome B (SSB), anti-Scl-70, and anti-Jo-1, was negative. At this time, pleurisy data are limited regarding *R. equi* infections in immunocompetent patients. However, the largest reported case series of *R. equi* infection in immunocompetent patients evaluated 19 patients with a broad spectrum of diseases from skin and soft tissue infections to invasive severe infections [3].

Treatment for *R. equi* infections includes a multitude of antibiotics, including macrolides, fluoroquinolones, aminoglycosides, rifampin, vancomycin, and linezolid [1]. Most literature agrees that the treatment regimen should include at least two antimicrobial agents to prevent resistance [1,10]. Additionally, if abscesses or an empyema form, drainage or surgical intervention is recommended [1]. Unfortunately, treating *R. equi* infection is difficult and often complicated by antimicrobial resistance, treatment failures, and clinical relapses. Therefore, a prolonged course of both intravenous and oral antimicrobial therapy is recommended by most expert opinions, especially in invasive, severe infections [4].

## Conclusions

Although most cases of *R. equi* occur in immunocompromised patients, it is important to keep this organism as part of the differential when dealing with infection in high-risk immunocompetent patients. When *R. equi* infects an immunocompetent patient, it is prudent to rule out any masked immunocompromised, as this may be the first presentation of a weakened immune system. What’s more, it is imperative to remember that *R. equi* frequently masquerades as a contaminant or other opportunistic organism. Therefore, communication between clinicians and the microbiology laboratory is essential to ensure accurate diagnosis, not only when there is a high index of suspicion but also when the patient shows no clinical improvement despite therapy. Our case is unique in that our patient had no immunocompromise or immunodeficiency, making him one of only a few immunocompetent patients to have been affected by *R. equi*, which adds to the literature that this opportunistic organism can cause significant disease in immunocompetent populations.
